# Quantification of maceration changes using post mortem MRI in fetuses

**DOI:** 10.1186/s12880-016-0137-9

**Published:** 2016-04-27

**Authors:** P. Montaldo, S. Addison, V. Oliveira, P. J. Lally, A. M. Taylor, N. J. Sebire, S. Thayyil, O. J. Arthurs

**Affiliations:** Centre for Perinatal Neuroscience, Imperial College London, Du Cane Road, London, W12 0HS UK; Institute of Child Health, University College London, London, UK; Great Ormond Street Hospital for Children NHS Foundation Trust, London, WC1N 3JH UK

**Keywords:** MRI, Autopsy, Pathology, Fetuses, Maceration, Intrauterine death

## Abstract

**Background:**

Post mortem imaging is playing an increasingly important role in perinatal autopsy, and correct interpretation of imaging changes is paramount. This is particularly important following intra-uterine fetal death, where there may be fetal maceration. The aim of this study was to investigate whether any changes seen on a whole body fetal post mortem magnetic resonance imaging (PMMR) correspond to maceration at conventional autopsy.

**Methods:**

We performed pre-autopsy PMMR in 75 fetuses using a 1.5 Tesla Siemens Avanto MR scanner (Erlangen, Germany). PMMR images were reported blinded to the clinical history and autopsy data using a numerical severity scale (0 = no maceration changes to 2 = severe maceration changes) for 6 different visceral organs (total 12). The degree of maceration at autopsy was categorized according to severity on a numerical scale (1 = no maceration to 4 = severe maceration). We also generated quantitative maps to measure the liver and lung T_2_.

**Results:**

The mean PMMR maceration score correlated well with the autopsy maceration score (*R*^2^ = 0.93). A PMMR score of ≥4.5 had a sensitivity of 91 %, specificity of 64 %, for detecting moderate or severe maceration at autopsy. Liver and lung T_2_ were increased in fetuses with maceration scores of 3–4 in comparison to those with 1–2 (liver *p* = 0.03, lung *p* = 0.02).

**Conclusions:**

There was a good correlation between PMMR maceration score and the extent of maceration seen at conventional autopsy. This score may be useful in interpretation of fetal PMMR.

## Background

Post mortem magnetic resonance imaging (PMMR) is increasingly used as an alternative to conventional perinatal autopsy [1–3]. PMMR along with additional minimally invasive investigations, is now considered as accurate as conventional autopsy and is particularly useful for cerebral, cardiac and abdominal imaging [4–7]. For these reasons, PMMR is now more increasingly offered worldwide to parents who refuse conventional autopsy [8]. Unlike conventional autopsy, PM MRI provides an interactive visualization and permanent archiving of three-dimensional data sets of internal organs [9]. Furthermore, internal organ volumes can be accurately estimated from the MRI data [10].

However, there are several changes observed on PMMR that are currently interpreted as relating to decomposition of the body, although these are poorly understood [11]. This is further complicated in fetuses, where there may have been a period of maceration prior to the delivery, as well as autolysis following the delivery. Maceration is the process where skin is softened and broken down within a fluid filled cavity, which will occur following fetal death within the amniotic fluid. Autolysis occurs after delivery and is the intracellular enzymatic breakdown of body tissues, although this can be slowed by cooling, refrigeration or even freezing. Both of these processes may have different effects on tissue appearances at autopsy or at post mortem imaging, and it is important to try to differentiate these effects in order to correctly interpret imaging findings.

The criteria for quantifying post-mortem changes and estimating post-mortem interval using conventional autopsy and histopathology are well established. This includes skin slippage, skin and umbilical cord discoloration, cranial collapse, level of mummification on external examination and loss of nuclear basophilia on histological examination [12–14]. Whilst these are estimates of intrauterine retention interval, they remain the current gold standard.

Understanding such PMMR artifacts is important not only for accurate clinical reporting, but also for the estimation of post-mortem interval, which may be of major clinical and medicolegal significance, particularly in stillbirth and in forensic cases. As most miscarriages and stillbirths occur between routine ultrasound scan appointments, which occur months apart, pinpointing the exact date of death on a clinical basis can be extremely difficult. In cases where PMMR is the initial investigation, information about the extent of maceration may inform about the need and/or utility for a full conventional autopsy and histopathological examination.

In this study, we hypothesized that some PMMR indices would correlate with pathological estimates of maceration, in particular fluid changes across different body organs.

## Methods

### Study Participants

This work was undertaken as a nested study in a large on-going prospective study—Magnetic resonance imaging autopsy study (MaRIAS) comparing post-mortem MRI with conventional autopsy in fetuses, newborns, infants and children [15]. We performed PMMR followed by conventional autopsy in a sequential cohort of fetuses referred to Great Ormond Street Hospital for Children and University College Hospital, London, between March 2007 and September 2011. The study had institutional approval (reference 04/Q0508/41) [16].

### Demographic details

We collected data on the date of birth/delivery, fetal gestation at time of delivery, time interval from delivery to MRI (imaging interval), and time interval from delivery to autopsy (post mortem interval) from maternal medical records.

### Imaging technique

**Fig. 1:**
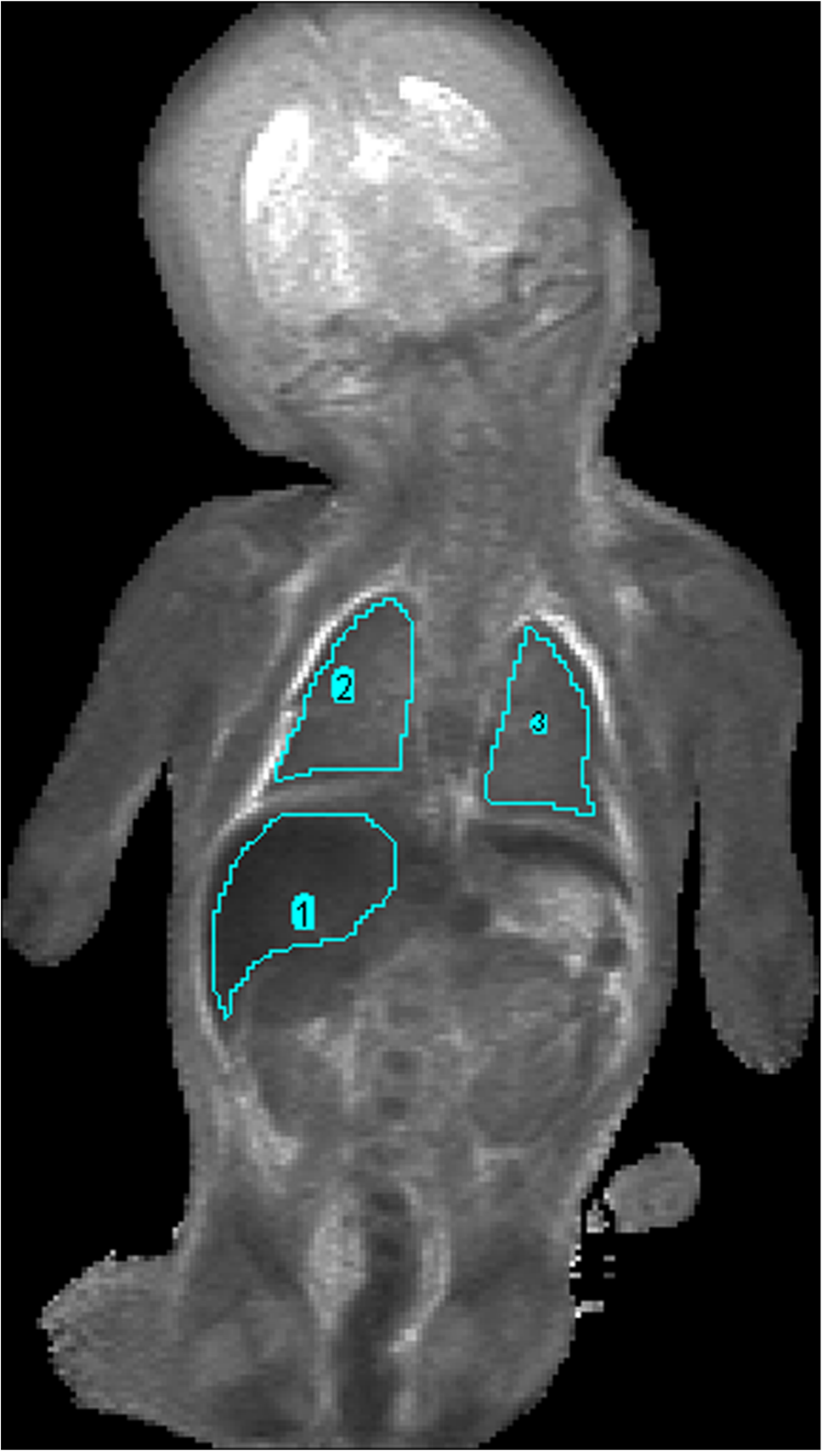
Voxelwise T2 values were generated from the model fit in each voxel and used to generate quantitative T2 maps, on which the lungs and liver were delineated

All scans were performed on 1.5 T MR scanner (Avanto, Siemens Medical Solutions, Erlangen, Germany) using 3D T2-weighted turbo spin echo (TSE), 3D T1-weighted volumetric interpolated breath-hold examination (VIBE) and 3D constructive interference in the steady state (CISS) [16]. The PMMR was performed as soon as practically possible; in most cases, this was 1–7 days after death. All fetuses were kept refrigerated at 4 °C prior to the MR imaging. In a subset of ten unselected fetuses we performed T2 relaxometry using an eight-echo turbo spin echo sequence (TR = 2400 ms, TE = 44,88,132,176,220,264,308,352 ms), with even echo images fitted to a mono-exponential decay function in MATLAB. T2 values were generated from the model fit in each voxel and used to generate quantitative T2 maps, on which the lungs and liver were delineated (Fig. 1). Mean T2 values within each region were calculated.

### Reporting MRI images

PMMR images were reported by an experienced Paediatric radiologist (OJA) with 7 years of experience, using the OsiriX platform (OsiriX Foundation, Geneva, Switzerland), masked to the age, gestation, imaging and post mortem interval of each case. Each of six body parts was visually scored for the degree of maceration, from 0 (no maceration) to 2 (severe maceration), giving a maximum total MRI score of 12. The body parts included brain (loss of grey/white matter differentiation), presence of subcutaneous oedema, pleural effusion, ascites, portal venous gas, and loss of abdominal organ soft tissue delineation. The loss of grey/white matter differentiation was based on visual evaluation based on prior experience and no atlas was used. A second reporter (neonatologist; ST) with 7 years of experience of PMMR also reported 42 cases, in order to give an index of inter-observer variability in MR scores.

### Reporting of autopsy data

Conventional autopsy was performed in accordance with national guidelines, [17] and reported by one of seven experienced perinatal or Paediatric pathologists, masked to the PMMR findings. Conventional autopsies were then retrospectively scored by a single pathologist (NJS) who had performed some of the conventional autopsies, according to the autopsy report and the images taken at the time of autopsy. The autopsy score was from 1 to 4 (none, mild, moderate and severe maceration), based on the external evaluation of the fetus including skin slippage, skin discoloration and overlapping of the skull structures. Each of these scores suggests an approximate time of retention of the fetus in utero after death (intrauterine interval).

### Statistical analysis

We examined the correlation between PMMR score and autopsy scores of maceration. We also correlated T2 values with gestational age using Pearson or Spearman rho test, based on the data distribution. We also examined area under the receiver operating characteristic curve (AUC) for PMMR scores to detect moderate-severe maceration as reported by autopsy (gold standard), and generated diagnostic indices with 95 % confidence intervals (exact statistics) for the optimal threshold value [18]. The sample was divided in two groups: moderate or severe maceration and no or mild maceration at autopsy. Mann Whitney *U* test was used for comparison of distributions between the two groups at a *p* < 0.05 significance level. We used SPSS Statistics 22 (IBM Corp.—U.S.A.) for statistical analyses**.**

## Results

### Demographic data

PMMR was available for assessment on 75 fetuses, including 15 miscarriages (gestation <22 weeks) and 60 stillbirths (gestation >22 weeks). Mean gestation age at delivery was 30.5 ± 8.2 weeks. Nineteen (25.4 %) of the 75 fetuses showed no evidence of maceration at autopsy (autopsy score 1), 20 cases (26.6 %) had mild maceration (autopsy score 2), 24 (32 %) had moderate maceration (autopsy score 3), and 12 (16 %) had severe maceration (autopsy score 4). The median time between MRI and autopsy was 1 day (range 0–7).

### Maceration/autolysis on post-mortem MRI

**Fig. 2:**
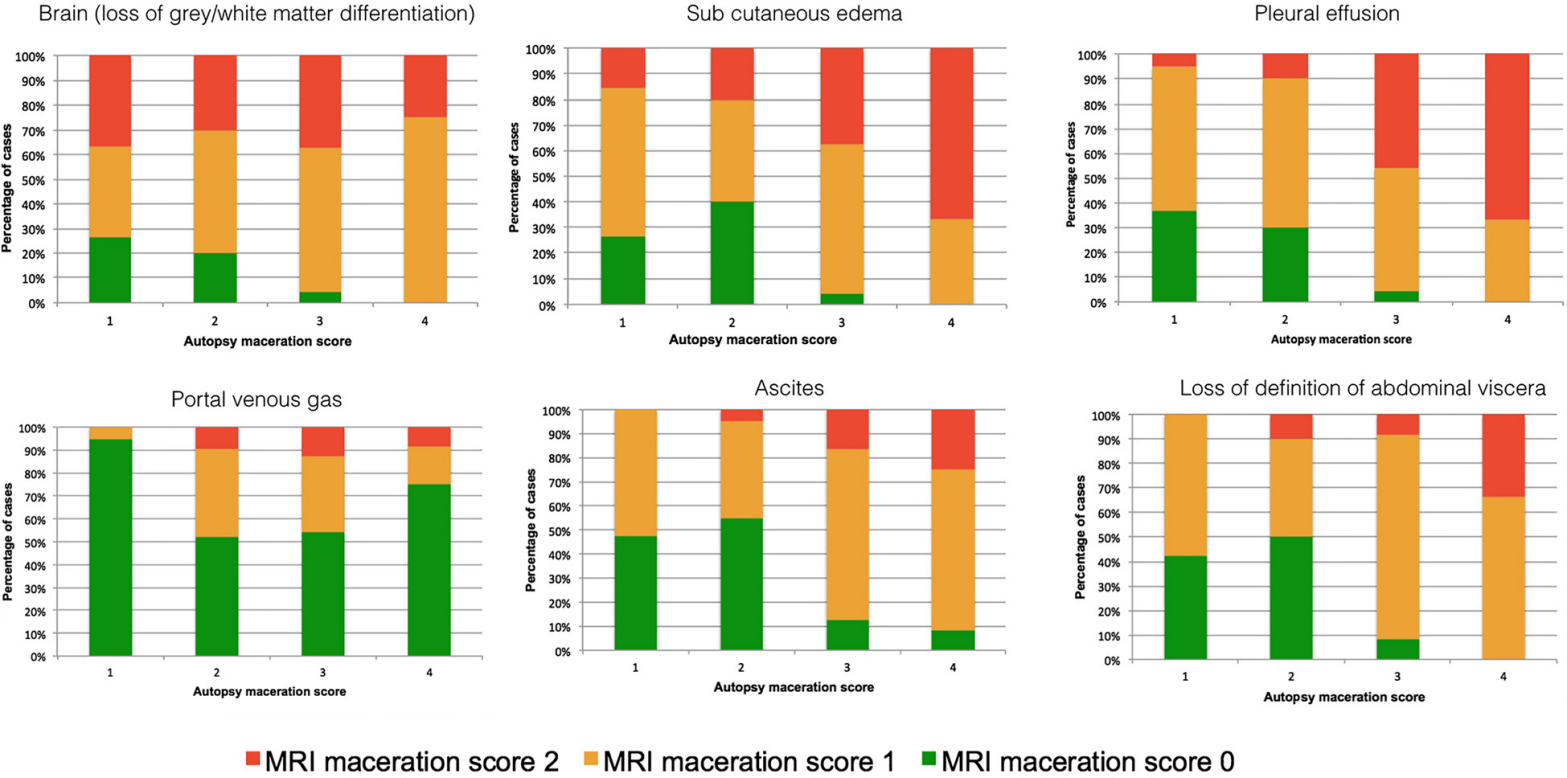
Post-mortem MRI maceration scores for individual organ systems compared with autopsy maceration grade

A good correlation was seen between the median PMMR maceration score and autopsy maceration score (*R*^2^ = 0.93). The maceration scores of all internal organs increased with increasing maceration noted at autopsy. All the fetuses who had a PMMR score 1–2 (moderate-severe maceration) on assessment of brain (loss of grey/white matter differentiation), subcutaneous oedema, pleural effusion, ascites, portal venous gas or abdominal organ soft tissue delineation, showed severe maceration at autopsy (score 4). Presence or absence of portal venous gas did not correlate well with the extent of maceration (Fig. 2). Inter-observer reproducibility was moderate, with mean difference between repeated imaging scores of 2.7 ± 1.8.

Overall, a PMMR score of 4.5 had a sensitivity of 91 % (95 % Confidence Intervals (CI) – 78 to 97) and a specificity of 64 % (95 % CI – 48 to 77) for predicting moderate or severe maceration at conventional autopsy.

**Fig. 3:**
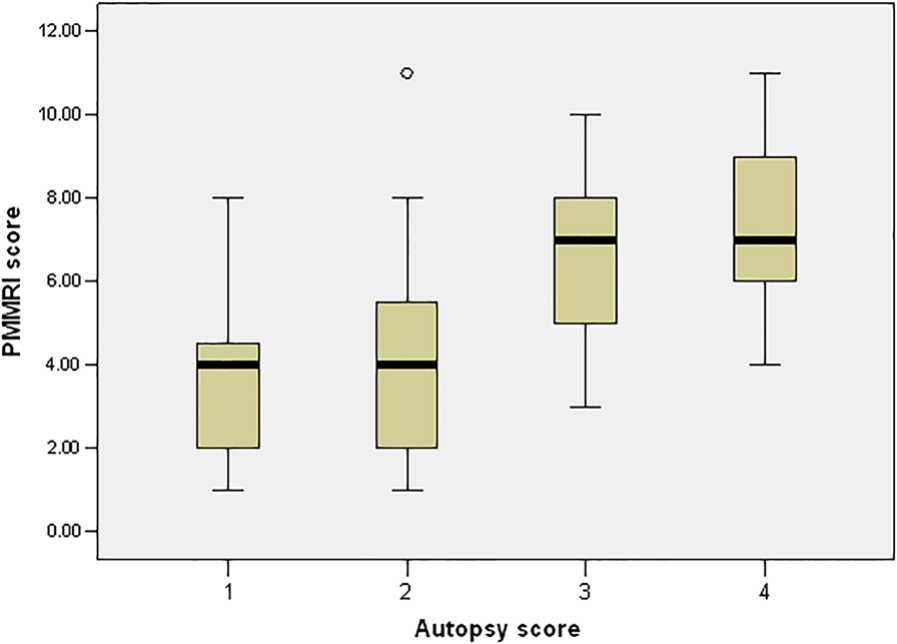
Median (min, max, Q1 and Q3) of overall post-mortem MRI maceration score compared with maceration score at autopsy

The median PMMR scores for the four different levels of maceration recorded at conventional autopsy are presented in Fig. 3. The median (interquartile range) PMMR score was significantly higher in cases with moderate or severe maceration at autopsy (group 3 and 4), as opposed to those who had no or mild maceration at autopsy (7 (5.8 to 8.0) and 4 (2.0 to 5.0) respectively, *P* < 0.001*)*.

### Postmortem Liver and Lung T_2_

**Fig. 4:**
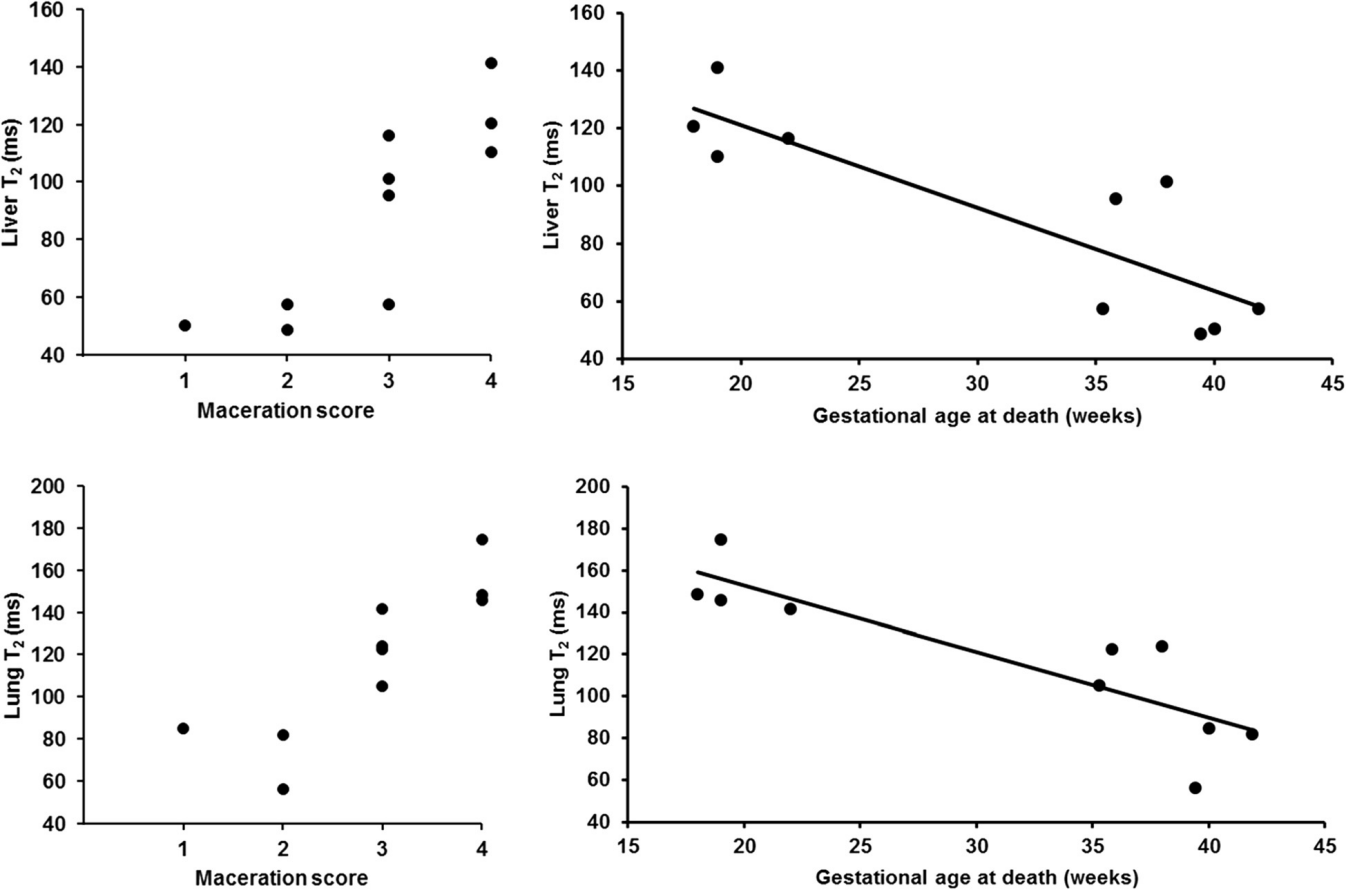
Correlation of liver and lung T2 with maceration score and gestational age Top Row: Liver T2 variation with maceration score (*left*) and gestational age at death (*right*). Bottom Row: Lung T2 variation with maceration score (*left*) and gestational age at death (*right*)

Liver and lung T_2_ were increased in fetuses with maceration scores of 3–4 (median (IQR): liver 110(20)ms, lung 142(24)ms) in comparison to those with 1–2 (liver 50(4)ms, lung 82(14)ms; liver *P* = 0.03, lung *P* = 0.02). However, T_2_ also correlated inversely to the gestational age at death (liver *R*^2^ = 0.72, lung *R*^2^ = 0.75) (Fig. 4).

## Discussion

Our data suggest that PMMR score correlates well with the extent of maceration seen at autopsy. A PMMR score ≥4.5 had a high sensitivity for detecting moderate or severe maceration at autopsy. Furthermore, increasing maceration is associated with the prolongation of T_2_ values in the liver and lungs. These preliminary PMMR findings are promising for establishing further imaging correlates of autopsy findings.

In particular, a reduction in the tissue contrast in the brain strongly correlated with the extent of maceration, but presence of portal venous gas did not. This is in agreement with a smaller pilot study of 11 fetuses by Victoria et al., who found that that loss of gray-white matter differentiation in the brain, the presence of pleural effusions and small lung volumes were suggestive of in utero fetal death at fetal MRI [19]. Appreciating the brain changes caused by maceration is particularly useful for accurate PMMR reporting, as PMMR has been shown to provide important clinical and diagnostic information in over 50 % of fetuses where conventional brain autopsy was non-diagnostic due to maceration and autolysis [6]. Portal venous gas instead, may better represent resuscitation related air redistribution, rather than decomposition, as has been shown on PMCT [20].

To our knowledge, this is the first preliminary study to assess whether quantitative MR measurements can be useful to identify the degree of maceration. External features of maceration are historically better indicators of early rather than late changes of maceration [13, 14]. Pathological estimates are therefore likely to be less specific at longer intra-uterine retention intervals (higher maceration scores). Maceration likely reflects a combination of cellular breakdown and tissue degradation, leading to changes in tissue structure and permeability and fluid redistribution throughout body compartments. Our data suggest that fluid redistribution in both lungs and liver, which occur with moderate-severe maceration, can be detected at PMMR, although gestational age changes may also contribute to the T2 signal seen.

There are limitations to this retrospective study of a small number of PMMR cases, although all had a full autopsy for comparison. Although our cases covered a broad range of maceration indices, the extent of maceration and autolysis varies depending of the exact intrauterine retention period after the fetal demise (which is rarely accurately obtainable), and storage conditions of the fetus after delivery. Although all fetuses were rapidly refrigerated after death and during transport, the period between delivery and refrigeration was not recorded. Furthermore, due to refrigeration, none of our fetuses was severely decomposed or putrefied, which may account for the low incidence of portal venous gas in this cohort. This type of imaging score may need modification where the body has been at room temperature for a prolonged period.

## Conclusion

The PM MRI maceration score presented in this work correlates well with the extent of maceration seen at conventional autopsy. This score may be useful in interpretation of fetal PMMR in future.

### Ethics approval and consent to participate

The study was approved by the GOSH-ICH research ethics committee (reference 04/Q0508/41). Parental consent was obtained for the MRI as stipulated by the ethics committee [21].

### Availability of data and materials

All relevant data supporting our findings are anonymised and stored at Great Ormond Street Hospital for Children.

## Abbreviations

AUC: area under the receiver operating characteristic curve
PM: post-mortem
PMMR: post-mortem magnetic resonance imaging
